# Accelerated Enveloping Distribution Sampling (AEDS) Allows for Efficient Sampling of Orthogonal Degrees of Freedom

**DOI:** 10.1021/acs.jcim.2c01272

**Published:** 2022-12-13

**Authors:** Oriol Gracia Carmona, Chris Oostenbrink

**Affiliations:** Institute for Molecular Modeling and Simulation, Department of Material Sciences and Process Engineering, University of Natural Resources and Life Sciences, Vienna, Muthgasse 18, 1190Vienna, Austria

## Abstract

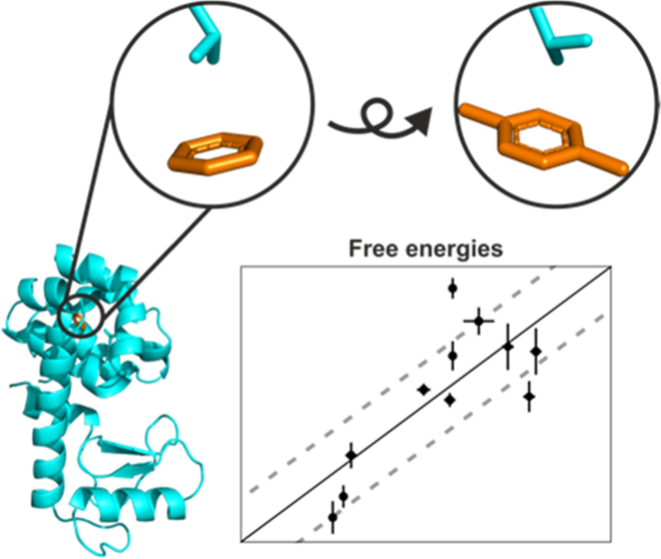

One of the most challenging aspects in the molecular simulation of proteins is the study of slowly relaxing processes, such as loop rearrangements or ligands that adopt different conformations in the binding site. State-of-the-art methods used to calculate binding free energies rely on performing several short simulations (lambda steps), in which the ligand is slowly transformed into the endstates of interest. This makes capturing the slowly relaxing processes even more difficult, as they would need to be observed in all of the lambda steps. One attractive alternative is the use of a reference state capable of sampling all of the endstates of interest in a single simulation. However, the energy barriers between the states can be too high to overcome, thus hindering the sampling of all of the relevant conformations. Accelerated enveloping distribution sampling (AEDS) is a recently developed reference state technique that circumvents the high-energy-barrier challenge by adding a boosting potential that flattens the energy landscape without distorting the energy minima. In the present work, we apply AEDS to the well-studied benchmark system T4 lysozyme L99A. The T4 lysozyme L99A mutant contains a hydrophobic pocket in which there is a valine (valine 111), whose conformation influences the binding efficiencies of the different ligands. Incorrectly sampling the dihedral angle can lead to errors in predicted binding free energies of up to 16 kJ mol^–1^. This protein constitutes an ideal scenario to showcase the advantages and challenges when using AEDS in the presence of slow relaxing processes. We show that AEDS is able to successfully sample the relevant degrees of freedom, providing accurate binding free energies, without the need of previous information of the system in the form of collective variables. Additionally, we showcase the capabilities of AEDS to efficiently screen a set of ligands. These results represent a promising first step toward the development of free-energy methods that can respond to more intricate molecular events.

## Introduction

Computer simulations can be used to answer biological questions, as well as to help guide experiments in the fields of protein engineering and drug design in a cheap and environmentally friendly way.^1^ In the field of drug discovery and development, computer simulations can help to assess possible hits by calculating binding free energies, which often correlate with the affinity of the studied compounds.^2,3^ That is why, over the years, there has been a huge effort to improve the methodologies used to calculate free energies, with the objective to arrive at a precision range useful for real case scenarios.^4−6^ One of the most common approaches is the use of molecular dynamics (MD) simulations together with alchemical perturbation methods, such as free-energy perturbation (FEP) or thermodynamic integration (TI).^7−12^ Those methods are based on performing multiple short simulations, in which the ligands of interest are progressively converted from one to the other based on a coupling parameter λ. This, together with the use of thermodynamic cycles, allows for the calculation of relative binding free energies between pairs of compounds without the need of performing individual simulations of the binding process of each of them, which would be more costly.^13^ It is also possible to calculate the full binding free energies using alchemical methods by perturbing the ligand into a dummy molecule that does not interact with anything.^14^ However, these kinds of simulations are often harder to converge, and in most of the scenarios, one is more interested in the free energy differences between compounds than the absolute ones, especially when dealing with congeneric series of ligands.

Despite all improvements, such as the use of the Bennett acceptance ratio (BAR) method to estimate free energies,^12,15^ there are still challenging topics that need to be addressed before free-energy methods can really be used broadly on any system.^6,16,17^ One example where free-energy methods struggle is the achievement of sufficient sampling of all of the relevant degrees of freedom, especially when there are slow processes, such as changes in the side-chain rotamers, backbone rearrangements, or active-site water displacements, among others.^17−22^ Those processes may have a high associated energy barrier, which requires more time to sample than the length typically used in this kind of simulation. This challenge becomes more pronounced with free-energy perturbation methods since they rely on performing multiple simulations, and one would need to sample the slow events in multiple simulations to have a well-connected pathway and account for the impact of these events on the free energy of binding. One solution could be to elongate each simulation to the point in which capturing the slow events reversibly is possible. However, that would make those techniques not useful for real applications due to the amount of computational time that is required.^23^ Alternatively, one can use enhanced sampling tools like replica exchange simulations to mix alternative conformations over all λ-values. This requires an efficient sampling of the orthogonal degrees of freedom in at least one replica, for which reason a higher local temperature may be applied.^24^ Another solution would be to add biases to the system, such that the slow degrees of freedom can be sampled in the time frame of the simulation length. These kinds of approaches have displayed success in several cases.^25−28^ Nonetheless, to define appropriate biases, previous knowledge of the system is required, which may not be available *a priori*.

**Figure 1:**
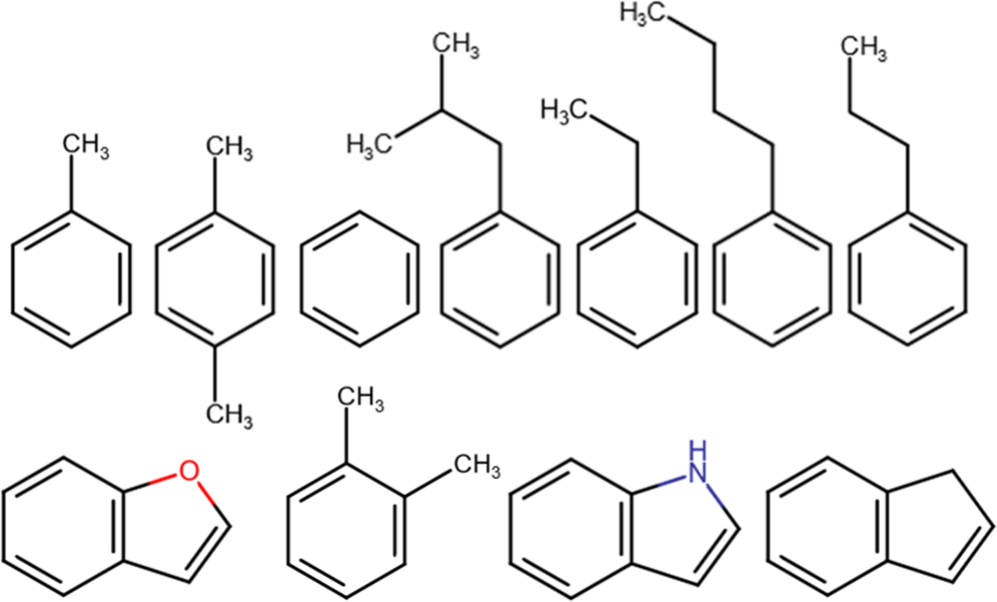
Subset of ligands that bind on the engineered apolar cavity of the T4 lysozyme L99A mutant, from left to right, top to bottom: toluene, *para*-xylene, benzene, isobutylbenzene, phenylethane, *N*-butyl-benzene, *N*-propyl-benzene, benzofurane, *ortho*-xylene, indole, indene.

An ideal test system to study this kind of slow process is the T4 lysozyme L99A mutant. T4 lysozyme is a small protein with an engineered apolar binding site, for which there is available binding data for several small compounds, see Figure 1.^29^ This protein has been extensively studied both experimentally and computationally.^29−37^ It is known that its engineered binding site contains a valine (valine 111), whose conformation influences the binding of some of the studied ligands, leading to errors up to 16 kJ mol^–1^ when the conformational preferences of this valine are not sampled properly.^32^ Additionally, it has been reported that some ligands induce a slight shift of a nearby helix, helix F, to be able to accommodate bigger ligands.^38−42^ The simplicity of the system and the ligands under study, together with the presence of these well-studied slow processes, makes the T4L an ideal test case to benchmark the performance of different free-energy methods.

One attractive alternative to the multistep alchemical methods is the use of a reference state, which is obtained from the combination of all of the states of interest, thereby allowing for sampling of the whole relevant conformational space of several ligands at the same time in a single long simulation. Examples are the one-step perturbation method with soft-core reference states^43^ or the enveloping distribution sampling (EDS) method.^44^ This simplifies the complexity of the sampling problem by decreasing the number of times that one needs to sample the slow orthogonal degrees of freedom. That is, instead of running multiple simulations for each pair of compounds, one can run a single, longer simulation. It is important to note that these techniques often struggle to sample conformations that are relevant for all of the ligands or endstates of interest due to high energy barriers that separate the different areas of the conformational space. In this context, accelerated enveloping distribution sampling, AEDS, is a recently developed technique that falls into the category of reference state methods.^45,46^ As in regular EDS, an energy offset is added to the Hamiltonian of each ligand of interest so that all of the energy minima are located at the same height and hence ensure roughly equal sampling. Furthermore, AEDS combines the use of a reference state with an acceleration potential, similar to the one used in Gaussian accelerated molecular dynamics (GAMD),^47^ to decrease the energy barriers between states and ensure better sampling. In GAMD, a single energy threshold is used to determine when the boosting potential should be added, moving the energy minima up to decrease the height of the energy barriers. In contrast, AEDS uses two energy thresholds (*E*_min_ and *E*_max_), and the boosting potential is only added within that region by pulling the energy maxima down. In this way, AEDS achieves a flatter energy profile without distorting the energy minima of interest. In a similar fashion to GAMD, the acceleration parameters for AEDS can be determined from a previous search run. While AEDS has been used for ligand-binding examples,^46^ it has not been tested yet in more complex systems, where slow orthogonal degrees of freedom are present. There is currently no knowledge on how such events affect the parameter search and whether AEDS affects the sampling of such slow processes.

In the present work, we tested the capabilities and performance that AEDS has when dealing with systems that have slow relaxing processes, using the T4 lysozyme L99A mutant as a test system. We assess the AEDS performance by examining both the free energies obtained and the sampling of the slow events with the advantage of not adding any kind of bias based on previous knowledge of the system. Additionally, we also provide guidelines on how to correctly use, set up, and analyze this technique.

## Methods

### System Preparation

The starting crystal structure for the T4 lysozyme was obtained from the protein data bank, PDB code 181L.^29^ The protonation states of the amino acids were assigned based on a pH of 7, using the side chain orientation and the H++ web server.^48^ All of the MD simulations were performed using the GROMOS simulation package (https://www.gromos.net),^49^ and the models were parametrized using the 54A8 GROMOS forcefield.^50^ The different ligands under study were parameterized using the Automated Topology Builder web server (ATB) and adjusted to better match the GROMOS charges.^51^ The python package SMArt^52^ was used to build the reference state and perturbation topologies using a single topology approach, which contains the necessary extra atoms for all of the endstates as dummy particles.^53,54^ To simplify the perturbation topology, the bond lengths of the five-member ring of indene, indole, and benzofurane remained constant.

Short energy minimizations were performed using the steepest descent algorithm in vacuum. The models were then placed in a periodic rectangular water box with simple point charge (SPC) water molecules,^55^ leaving a minimum distance of 1.4 nm from the solute to the box walls. The systems were further minimized using the steepest descent algorithm with position restraints on the solute atoms.

Counterions were added by replacing water molecules with the most favorable electrostatic potential to reach charge neutralization using the program ion provided in the GROMOS++ package.^56^ All of the replicas for each model were generated by setting different initial velocities sampled from a Maxwell–Boltzmann distribution at 60 K. The systems were thermalized up to 300 K by five discrete steps with position restraints on the solute atoms. The strength of the restraints was decreased by a factor of 10 from 2.5 × 10^4^ to 0 kJ mol^–1^ nm^–2^.

Production simulations of 10 ns each were performed at a constant temperature of 300 K and a constant pressure of 1 atm using the Nosé–Hoover chain algorithm for the temperature control with 5 chains^57^ and the weak coupling algorithm for the pressure,^58^ with a coupling time of 0.5 ps and an estimated isothermal compressibility of 4.575 × 10^–4^ kJ^–1^ mol nm^3^. Newton’s equations of motion were integrated using the leapfrog algorithm with a time step of 2 fs. The SHAKE algorithm^59^ was used to maintain the bond lengths at their optimal values. Long-range electrostatic interactions beyond a cutoff of 1.4 nm were truncated and approximated by a generalized reaction field^60^ with a relative dielectric permittivity of 61.^61^ Nonbonded interactions up to a distance of 0.8 nm were computed at every time step using a pairlist that was updated every 10 fs. Interactions up to 1.4 nm were computed at pairlist updates and kept constant in between. To avoid the ligand from diffusing away from the active site, weak distance and dihedral restraints were used, with a force constant of 125 kJ mol^–1^ nm^–2^ and 0.0508 kJ mol^–1^ rad^–2^, respectively. Special care was used to avoid influencing the degrees of freedom of interest with the defined restraints. The impact of the restraints was accounted for during the reweighting process. More details of the restraints used can be found in the Supporting Information.

### AEDS Simulations

The EDS reference state, *H*_R_, is built by combining the Hamiltonian of the different states, *H*_i_, using an energy offset, Δ*F*_i_^R^, to place all of the energy minima at the same level and ensure a more equal sampling of all of the states (eq 1)^44^
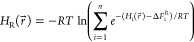
1with *RT* being the gas constant multiplied by the absolute temperature. The resulting energy landscape can have high energy barriers between the states that can hamper correct sampling of all of the states of interest. To solve this issue, AEDS uses a harmonic boosting potential defined as follows^45^
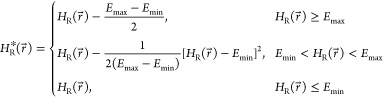
2where *E*_max_ is the energy maximum found when crossing between states and *E*_min_ is a parameter to define the range of energies to accelerate. The required parameters (*E*_max_, *E*_min_, and the offsets) are obtained using a search algorithm.^46^ First, a complete search of 10 ns is performed on the ligands in solution. For the simulations of the protein–ligand complex, the same *E*_max_ and *E*_min_ found during the search in solution are used, but an additional 10 ns simulation is performed to update the offsets. In the search runs, the offsets are estimated from the free-energy differences between the accelerated endstates, using the perturbation formula. For the ensemble average in this approach, a linearly increasing memory window is used, going from 1000 steps (2 ps) to 10,000 steps (20 ps). By doing the search runs in this way, one can achieve better convergence with less simulation time.^46^ Once the correct parameters are obtained, a final AEDS production run is performed. The AEDS simulations use the same simulation parameters as the conventional MD performed with the addition of the AEDS algorithm.

## Simulation Analysis

The dihedral angle sampling, atom-positional root-mean-square deviation (RMSD), and root-mean-square fluctuations (RMSF) are computed using the GROMOS++ analysis tools.^49^ The free energy of binding is calculated using the GROMOS++ *dfmult* program.^49,62^ The effect of the restraints is accounted for by including them in the reference state energy prior to the calculation of the free energies with *dfmult*. To avoid a bias by selecting a single ligand as a reference for all pair differences, the reference AEDS state was used instead to anchor the relative ΔG to the experimental data. To simplify the comparison of the AEDS results to the experimental ones, the obtained free energies are shifted from the arbitrary range relative to the reference state and to the experimental one by adding a constant. This shift is calculated as the average of the difference between the experimental free energy and the ΔΔ*G* to the AEDS reference state. The degree of freedom lost in the process is accounted for when computing the reported statistics by subtracting one degree of freedom from the calculation.

## Results

### AEDS Parameter Search

To determine the acceleration parameters required for AEDS (*E*_max_, *E*_min_, and offsets) an initial search simulation of 10 ns of all of the ligands in Figure 1 in solution was performed. During this initial search, all of the acceleration parameters were allowed to fluctuate at the same time to account for their respective effect on each other. To allow for rapid fluctuations at the beginning of the search and smaller ones toward the end, a linearly increasing memory decay function was used.^46^ This memory decay function should be broad enough to account for any relevant slow event of interest.

A closer examination of the search run revealed that both *E*_max_ and *E*_min_ converged during the first 4 ns. The remaining 6 ns in which *E*_max_ and *E*_min_ no longer changed significantly was used to estimate the energy offsets. The energy offsets do not converge as smoothly as the energy minima and maxima but oscillate around their optimal values (see Figures S2–S5 in the Supporting Information). One can take the average, but a better approach is to first filter the value spikes that sometimes appear when one state is not being sampled for a while and then computing the average. The obtained parameters from the search run can be found in Table 1.

**Table 1:** Acceleration Parameters, in kJ mol^–1^, of the Ligands in Solution (Unbound Ligand), the Ligand Bound to the Protein (Bound Ligand), and the Ligand Bound to the Protein but with the Offsets Recalculated Using the Production Run (Adjusted Offsets)^a^

	unbound ligand	bound ligand	adjusted offsets
*E*_max_	64.54	64.54	64.54
*E*_min_	–435	–435	–435
offsets (Δ*F*_i_^R^)			
indene	0	0	0
indole	–4.1	–10.2	–9
benzofurane	–20.5	–30.0	–28
benzene	–61.6	–66.0	–63
*ortho*-xylene	11.1	21.3	21
*para*-xylene	9.0	11.2	9
toluene	–12.8	–23.0	–20
*N*-propyl-benzene	38.9	41.4	37
ethylbenzene	22.0	20.6	20
isobutylbenzene	62.8	52.5	53
*N*-butyl-benzene	55.4	45.7	45

a The offset for indene is 0 kJ mol^–1^ in all three cases because it is the one that was used as a reference to calculate the remaining ones, as described in ref (46).

After the initial search run, an additional search run of 10 ns for the protein–ligand complex was performed. This time only the offsets were allowed to fluctuate, and the *E*_max_ and *E*_min_ values were kept constant, using the values that were estimated during the search run of the ligands in solution. The *E*_max_ and *E*_min_ values obtained during the search in solution are already a close estimation for the acceleration range in the protein–ligand system, and by keeping them constant, a faster convergence of the energy offsets can be achieved.^46^ After all of the acceleration parameters were estimated (Table 1), a production run of 10 ns for both the ligands in solution and in complex with the protein was performed.

To assess the quality of the obtained simulation, one can calculate how many transitions between states happen during the simulation, how much time each state is sampled, and how many frames contribute to the free energy of each endstate. The sampled endstate for every time point was defined as the endstate that has the lowest energy at that given time.^46^ However, a more accurate way of computing the sampling times would be to use the relative relevance (weights) of the different states over all of the simulation frames. In this way, one also takes into account those snapshots in which more than one state had favorable energies instead of assigning all of the weight to the state with the lowest energy. The weight of each endstate can be calculated with eq 3, and the obtained values can be normalized and expressed as a fraction for every molecule.
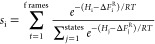
3The amount of frames that contribute to the free energy of each compound can be estimated by counting how many frames the energy difference between the reference state and the endstate are below their free-energy difference plus *RT*.^63^

The most reliable free-energy estimates will be obtained if there are several transitions between states during the simulations and equal sampling of all of the endstates (Table 2). However, in practice, this is quite difficult to achieve, and as long as there is at least reasonable sampling of all of the endstates, and enough frames contributing to the free energy, the free energies that one can extract from those simulations will be accurate enough. The more similar the endstates are between them, the less equal sampling time is required, since their relevant conformational space will be similar, making the energies that are obtained during the sampling of a different endstate still relevant for the remaining endstates. This can be seen especially for the small compounds (such as benzene and toluene). For these small endstates, the conformations sampled by the bigger endstates in the protein are also relevant for their free energy, leading to a larger number of contributing frames.

**Table 2:** Number of Transitions, Sampling Time, and Frames Contributing to the Free Energy (in %) of the System with Only Solvent (Unbound Ligand), the Ligand Bound to the Protein (Bound Ligand) and the Ligand Bound to the Protein but with the Offsets Recalculated Using the Production Run (Adjusted Offsets), Averaged over the Replicates

	unbound ligand	bound ligand	adjusted offsets
transitions	11,266	6166	7298
sampling times^a^			
indene	10.5%/7.1%	9.2%/5.7%	11.3%/6.5%
indole	10.1%/4.3%	6.5%/4.1%	8.9%/5.8%
benzofurane	8.2%/3.2%	2.3%/6.9%	6.6%/7.9%
benzene	11.0%/9.6%	3.2%/7.1%	5.3%/11.1%
*ortho*-xylene	12.2%/7.0%	16.3%/7.1%	23.3%/7.7%
*para*-xylene	12.5%/6.1%	15.0%/7.2%	7.6%/3.3%
toluene	7.6%/15.3%	2.3%/16.8%	4.5%/15.6%
*N*-propyl-benzene	4.0%/6.8%	31.5%/13.7%	10.0%/8.5%
ethylbenzene	10.4%/7.5%	7.6%/13.6%	12.0%/8.8%
isobutylbenzene	8.9%/5.1%	2.0%/1.3%	5.5%/3.7%
*N*-butyl-benzene	4.6%/3.0%	4.2%/2.8%	5.0%/3.4%

a On each column, the first number corresponds to the relative sampling times of each endstate, and the second number corresponds to the percentage of frames contributing to the free energy.

The production run in water displayed a large number of transitions and fairly equally distributed sampling times, ranging from 4% (*N*-propyl-benzene) to 12.5% (*para*-xylene), with a standard deviation of slightly less than 3 kJ mol^–1^. However, this was not the case for the production run in protein, where some endstates (benzofurane, toluene, isobutylbenzene) were undersampled, with less than 3% of the total simulation time, while the endstate *N*-propyl-benzene was oversampled, with more than 30% of the total simulation time. This can occur when not all of the minima have been correctly sampled during the search run, leading to slightly wrong offset parameters and trapping the simulation in those minima. Extending the search run could eventually allow the offsets to correct themselves, but a faster approach is to recalculate the offsets using the production run by applying the same formula as during the search,^46^ but using the data of the production run instead. After readjusting the parameters (Table 1), a new 10 ns production simulation was performed and the sampling was more equally distributed among all of the endstates and more transitions were observed during the simulations.

## Valine 111 Sampling

**Figure 2:**
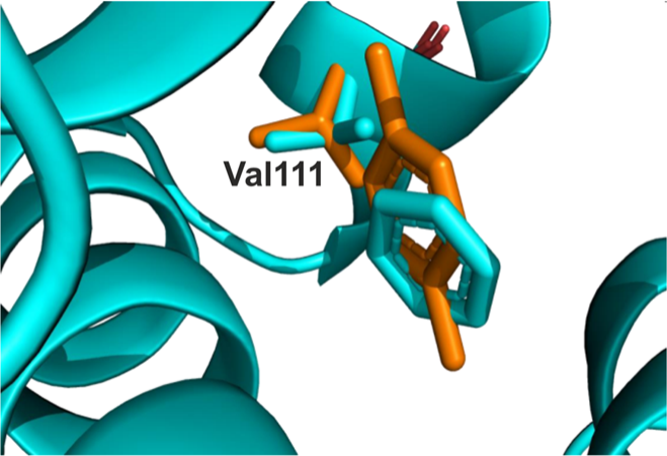
Binding pose to the T4L L99A lysozyme of the ligands, benzene (cyan) and *para*-xylene (orange). Shown is valine 111, which takes a different conformation to accommodate bulkier ligands as the *para*-xylene.

One of the challenging features of the T4 lysozyme L99A is the presence of a valine (Val111) in the active site, the orientation of which greatly influences the binding of some of the studied ligands.^29^ In most of the crystal structures, the χ_1_ dihedral of valine 111 is found in the trans conformation (180°), which is also the expected predominant state of the valine when the protein has no ligand bound in the active site. On the other hand, the crystal structure of some of the ligands displayed valine 111 with χ_1_ in the gauche conformation (−60°), which makes the interactions with those ligands more favorable.^29−31^ The *para*-xylene ligand is one of the most well-known cases of this, see Figure 2. Additionally, this valine is known to be fluctuating between the two conformations continuously. Failing to correctly sample this dihedral rearrangement has led to errors on the predicted free energies of up to 16 kJ mol^–1^.^32^

To assess the effect that AEDS has on the sampling of Val111, the conformation sampled during the AEDS simulations was compared with the sampling obtained from independent conventional molecular dynamics simulations of the protein without and with *para*-xylene bound to the active site. A total of four molecular dynamics simulations of 10 ns each were performed for each condition.

During the simulation without the ligand, the predominantly sampled conformational state of valine 111 was the trans conformation. On average, 5.5 transitions were observed per simulation and 80.5% of the total simulation time was spent in the trans conformation, which matches the expected dihedral conformation for the protein without any ligand bound. The simulations with *para*-xylene bound to the active site displayed a similar number of transitions, 5.5 per simulation, and predominantly sampled the gauche conformation, 83.8% of the total simulation time.

On the other hand, the AEDS simulation displayed a higher frequency of transitions compared to both the simulation with the ligand bound and the apo protein, with an average of 10.8 transitions per simulation, and sampled both conformations of interest, with similar sampling times for both conformations. The obtained average sampling time for each conformation was 45.6% for the trans conformation, 51.9% for the gauche conformation, and the remaining time in gauche+. Even though no explicit bias on the sampling of the Val111 χ_1_ angle was added, the use of an EDS reference state with acceleration and the resulting ligand shifting between the different endstates seems to have helped pushing the valine to go from one conformation to the other, leading to a more balanced sampling of both conformations of interest.

**Figure 3:**
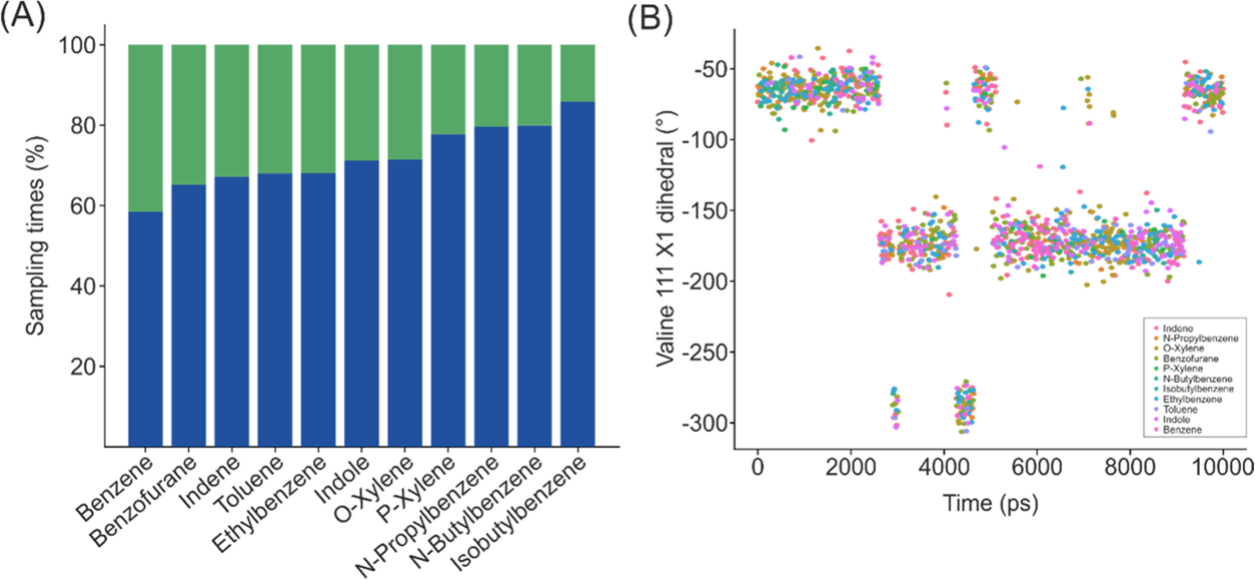
(A) Reweighted percentage of the total sampling time for the Val111 χ_1_ dihedral conformations to the endstate of each compound. Trans conformations in green and gauche in blue. The bulkiest endstates, whose preferred binding pose has the valine in the gauche conformation also displayed a higher percentage of their sampling time on that conformation compared to the smaller endstates, such as benzene and toluene. (B) Time series of valine 111 χ_1_ dihedral with the state being sampled at every given time as the color. For clarity, only every 15-th data point of one of the simulations is shown. The sampling of the different endstates was evenly distributed during the 10 ns of simulation time for all of the AED simulations.

To further inspect the sampling of Val111 with different endstates, the state being sampled was correlated with the observed dihedral conformation of Val111 at every given time. See Figure 3B for a time series of χ_1_ in which the markers are colored according to the compound with the lowest potential energy in the EDS reference state. For each of the ligands, both relevant conformations of Val111 are observed. Furthermore, those endstates, whose preferred binding pose includes Val111 in gauche conformation, namely, *para*-xylene, *n*-butylbenzene, isobutylbenzene, *n*-propylbenzene, and *ortho*-xylene, also had more sampling time of that given configuration compared to the other endstates. The average sampling times were reweighted for each compound based on the state energies (Figure 3A). The reweighting reproduces the trend in the preferred conformations with respect to the size of the ligand, as well as shows that gauche conformations are not improbable for the smaller compounds. Notably, AEDS did not only enhance the sampling of Val111, but, additionally, the sampling went hand in hand with the conformational preferences of every ligand.

### Helix Displacement

Another challenging feature of this system is the displacement that occurs on helix F, which is the helix composed of residues 107–115. This displacement occurs when a larger ligand binds the active site, resulting in an enlargement of the active site so that it can accommodate those ligands. Based on the electron density of the deposited crystal structures, this helix is known to be in three different conformations referred to as closed, intermediate, and open.^39,41^ The differences between the conformations of helix F are rather small, with atom-positional root-mean-square deviations (RMSDs) ranging from about 1.8 to 3 Å, while helix configurations belonging to the same group have RMSDs below 1 Å.^41^

This displacement is expected to be slower than the transition of valine 111 and thus harder to sample. To observe the effect of AEDS in this slow process, the movement of the aforementioned helix was monitored using as reference the crystal structures in complex with benzene for the closed conformation (pdb 4W52), *n*-butylbenzene (pdb 4W57) for the intermediate, and *n*-hexylbenzene (pdb 4W59) for the open one.^41^

**Figure 4:**
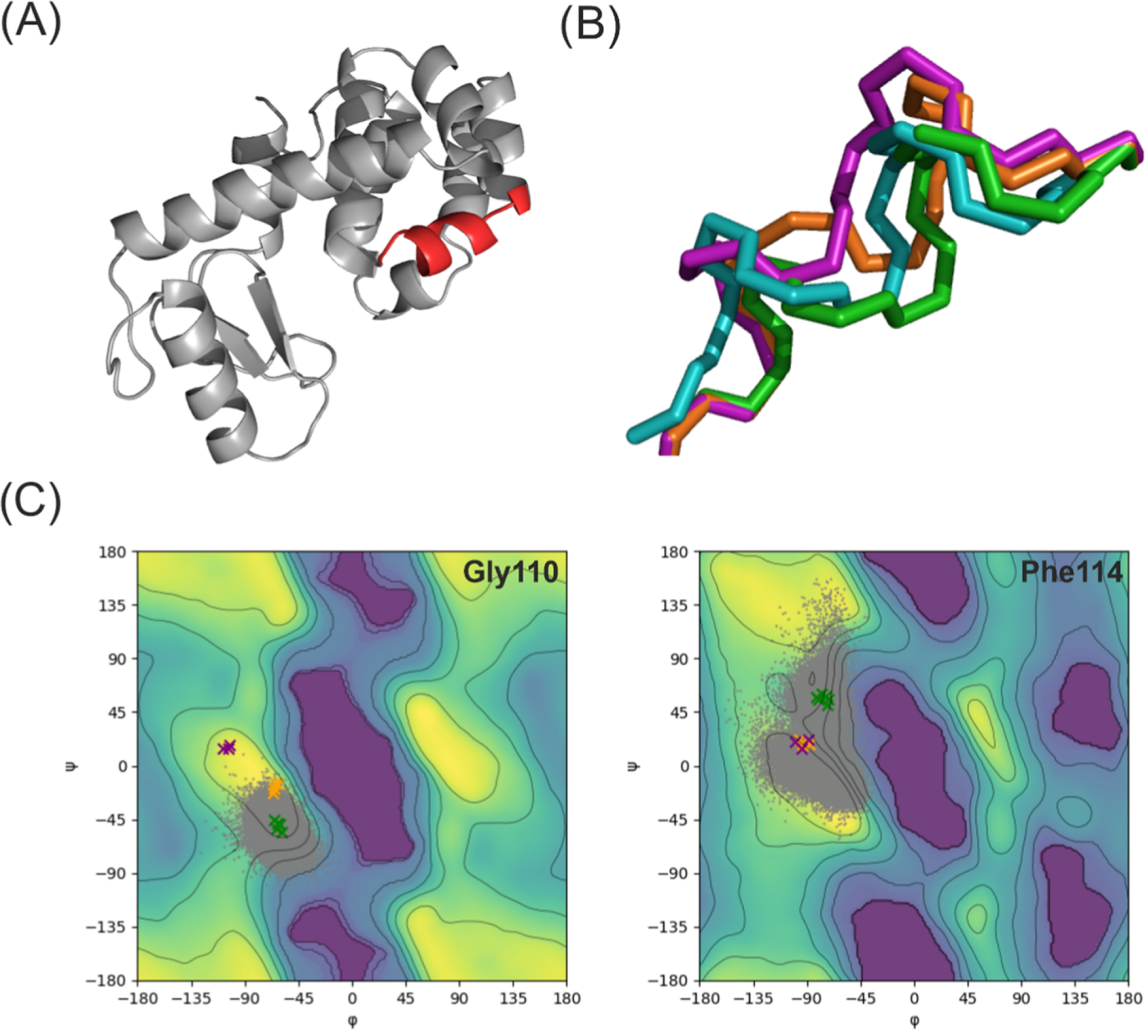
(A) Crystal structure of T4L L99A with helix F highlighted in red (PDB code: 181L ^29^). (B) Close up of the backbone orientation of helix F, closed conformation in green, intermediate conformation in orange, open conformation in purple, and average conformation during the AEDS simulations in cyan. The average conformation observed during the AEDS simulations contains both features of the closed and intermediate conformation. (C) Ramachandran plots for two of the characteristic residues of helix F. The green crosses depict the dihedrals for the closed conformation, the orange crosses for the intermediate conformation, and the purple crosses for the open conformation, as observed in the experimentally solved structures. The gray dots represent the dihedrals observed during the AEDS simulations. The background of the figures depicts the expected dihedral distribution for the Ramachandran plot of that residue,^64^ with yellow being the most probable configurations and purple the least probable ones. The distributions for the rest of helix F residues can be found in Figure S8.

The average position of helix F during the simulation is a middle point between the closed and intermediate conformations (Figure 4 panel B), and the RMSFs of the backbone atoms are higher than the differences that one observes in the crystal structures, S7. Helix F also sampled conformations different from the ones seen in the crystal structures, making the task of classifying individual snapshots based on RMSD difficult. The observed conformation of helix F was assessed by aligning each snapshot to the references and calculating the RMSD. The RMSD calculations showed similar results, revealing that the conformation of helix F was neither the closed nor the intermediate one, but one that fluctuated between these. Other defining characteristics of this helix F are the ϕ and ψ dihedral angles of its residues. Figure 4C shows the sampling of these dihedral angles for Gly110 and Phe114 in gray. The ϕ and ψ angles as observed in PDB structures 4W51-9 are indicated as reference values for the closed, intermediate, and open states.^41^ The dihedral angle analyses show similar results as the RMSD study. The AEDS simulations sampled the dihedral space characteristic of the closed and intermediate conformations but not the dihedral space of the open one.

The only helix F conformation that was clearly not sampled during the simulation was the open one, which is the least relevant conformation for the ligands under study, with only the *iso*-butyl-benzene and the *n*-butyl-benzene crystal structures having that conformation present in their electron density data.^41^ Note, however, that even for those ligands, the intermediate configuration is the preferred one.

To further test if the acceleration parameters correctly captured this slow process and to test the independence of AEDS from the initial starting structure, an additional simulation was performed starting from a configuration obtained from the crystal of *n*-butyl-benzene instead of benzene. The same acceleration parameters (Table 1; adjusted offsets) were also appropriate for this different starting structure. The obtained free energies showed a 1.5 kJ mol^–1^ root-mean-square difference from the previous simulations and were within the uncertainty range of the free energies obtained starting from a crystal of the closed conformation (see Table 3 below). Finally, the preference of each endstate to each of the conformations of helix F was estimated by reweighting the obtained RMSD profiles although no significant difference was observed (Table S1 in the Supporting Information).

**Table 3:** Binding Free-Energy Differences in kJ mol^–1^ with Their Standard Error in Brackets, Their Respective Root-Mean-Square Error (RMSE), Slope of the Linear Regression, and Correlation Coefficient (*R*^2^)^a^

	Δ*G*_bind_(kJ mol^–1^)			
	experiment	AEDS full	AEDS groups	AEDS helix
indene	–22.84 (0.04)	–21.2 (0.9)	–21.9 (0.7)	–24.2
indole	–20.46 (0.25)	–20.7 (1.4)	–20.9 (1.2)	–20.7
benzofurane	–22.84 (0.12)	–17.3 (0.6)	–18.1 (1.0)	–18.5
benzene	–21.71 (0.67)	–19.2 (0.8)	–19.7 (0.9)	–17.2
*ortho*-xylene	–19.25 (0.25)	–20.9 (1.4)	–19.8 (0.6)	–21.9
*para*-xylene	–19.54 (0.25)	–23.5 (0.9)	–22.6 (0.2)	–23.2
toluene	–22.97 (0.25)	–23.7 (0.4)	–23.5 (0.4)	–23.3
*N*-propyl-benzene	–27.57 (0.08)	–29.4 (0.7)	–28.4 (0.1)	–26.7
ethylbenzene	–24.10 (0.29)	–23.1 (0.4)	–22.8 (1.0)	–22.3
isobutylbenzene	–27.24 (0.25)	–27.0 (0.8)	–28.3 (0.4)	–28.3
*N*-butyl-benzene	–28.03 (0.08)	–30.6 (1.0)	–30.6 (0.6)	–30.0
RMSE		2.7	2.2	2.7
slope		1.02	1.1	0.91
*R*^2^		0.59	0.72	0.54

a Experimental values are extracted from refs (30, 33). Estimated values from the four independent AEDS runs with all of the 11 endstates denoted as “AEDS full.” Estimated values from the AEDS runs divided into subgroups based on chemical similarity denoted as “AEDS groups.” Estimated values from the AEDS run starting from an intermediate helix F conformation denoted as “AEDS helix.” The AEDS free energies are calculated as ΔΔ*G* from the reference state and then shifted to the experimental values, the lost degree of freedom is accounted for when calculating the RMSE.

### AEDS Free Energies

The free energies for all of the AEDS runs can be found in Table 3. The free energies obtained are in good agreement with the experimental free energies^30,33^ with a root-mean-square error (RMSE) of 2.7 kJ mol^–1^. The free energy results obtained by starting from a configuration with a different helix orientation also yield similar binding free energies within the same error range 2.7 kJ mol^–1^ RMSE, showcasing that the obtained free energies are independent of the selected starting structure (column AEDS helix). As expected, the simulation was obtained from the unrefined acceleration parameters, in which not all of the compounds were evenly sampled and were slightly less accurate than the other simulations, 3.1 kJ mol^–1^ (Supporting Information). This showcases the importance of obtaining correct sampling and enough transitions of the endstates of interest, a metric that can be used to assess how reliable are the free energies obtained for each of the endstates under study.

One reason that may have led to nonoptimal acceleration parameters is the difference of relevant conformational space between the different ligands. To simplify the sampling challenges, the endstates were divided into three subgroups of about four endstates each, sharing a common endstate, ethylbenzene, to then be able to combine all of the results together. The resulting groups were indene, indole, benzofurane, and ethylbenzene for group 1, benzene, toluene, *ortho*-xylene, *para*-xylene, and ethylbenzene for group 2, and *N*-propyl-benzene, *N*-butyl-benzene, isobutylbenzene, and ethylbenzene for group 3. These subgroups were selected based on the similarity of their energy landscapes. This was achieved by assessing the overlap of their energy distributions when sampling each of the other endstates. The subgroups obtained with this approach were similar to the ones that would result from a classification approach based on chemical similarity. This suggests that the chemical similarity between the ligand suffices to group them without needing to check their respective energies. After creating these subgroups, one new search run in bulk water and in protein was performed for each subgroup of endstates, following the same procedure as explained before.

**Figure 5:**
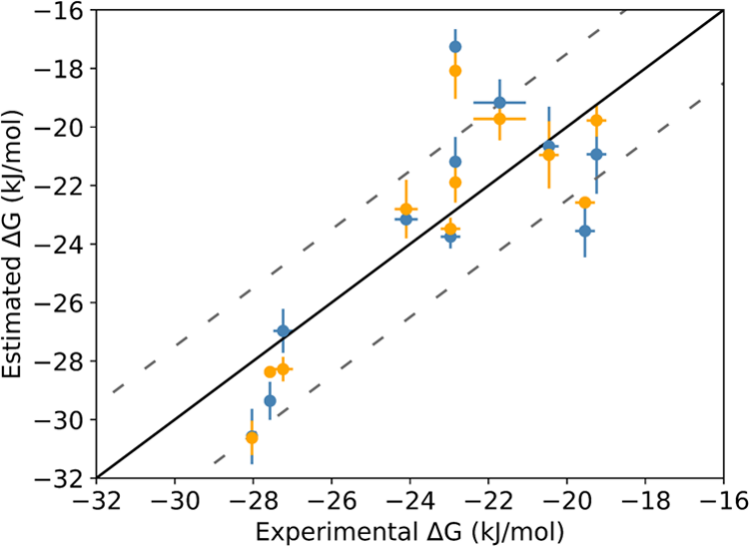
Binding free energies obtained from the AEDS simulations with all of the endstates (blue) and from the AEDS simulations divided into subgroups based on their chemical similarity (orange). The error bars indicate the standard errors, estimated from four independent AEDS simulations for the estimated Δ*G* and extracted from ref (29) for the experimental ones. The black diagonal line shows the perfect correlation and the dashed gray line shows the ±*RT* range. The free energies obtained from the runs with all of the endstates have a slope of 1.02, a correlation coefficient *R*^2^ of 0.59, and an RMSE of 2.7 kJ mol^–1^. On the other hand, the free energies obtained from the runs divided by subgroups have a slope of 1.1, a correlation coefficient *R*^2^ of 0.72, and an RMSE of 2.2 kJ mol^–1^.

For each of the subgroups, the search run alone was enough to correctly estimate the acceleration parameters, and no further refinement was required. After the parameter search, four independent production runs for each of the groups were performed. The obtained RMSE, 2.2 kJ mol^–1^, was lower than for the runs with all of the endstates, suggesting that this approach improves the sampling of the endstates (Table 3). The acceleration together with the obtained free energies for each subset of compounds can be found in the Supporting Information. Another advantage of this approach was that the runs required a smaller acceleration potential, which perturbed less the real energy landscape, decreasing the statistical inaccuracies that one faces during the reweighting process. This can be observed in the standard errors of the individual compounds, which are overall lower than the ones obtained from the AEDS runs with all of the states (AEDS full). The final obtained correlation coefficient (*R*^2^) was 0.59 for the run with all of the endstates and 0.72 for the runs in which the endstates were divided into three subgroups. The slopes were 1.02 and 1.1, and the RMSE was 2.7 and 2.1 kJ mol^–1^ respectively, indicating that both setups lead to good linear correlations with the experimental energies (see Figure 5). Although most of the slope and correlation coefficient steams from the difference between the strong and weak binders, the deviation from the experimental values for the endstates under study is very close. Note that in a real case scenario, one is interested in predicting the binding free energy within say 2.5 kJ mol^–1^ of the experimental value and not in precisely ranking compounds that differ by less than 2.5 kJ mol^–1^. Both the AEDS run with the 11 endstates and the ones divided by subgroups yield quite similar free energies, showcasing that the free-energy simulations were properly converged. For both cases, the highest source of error is the endstate benzofurane. This may indicate a problem on the forcefield and parametrization used for this compound rather than a lack of sampling since this endstate was among the most visited ones during the simulations.

### AEDS Screening

The previous sections show that accurate free energies of multiple states can be achieved with AEDS when adequate sampling is achieved. However, for some applications, such as during the virtual screening process or in the early stages of hit-to-lead development, knowing that the ΔΔ*G*_bind_ for all pairs of compounds may not be necessary since one is often interested only in the best binders among them. State-of the-art free-energy methods usually waste a significant amount of computational time sampling compounds that are not relevant. We hypothesized that AEDS could be used to circumvent this issue. Therefore, we explored if the acceleration parameters found in water are sufficient to identify the most promising compounds in the protein, without aiming for accuracy in the free-energy estimates. In this scenario, one can run a production of AEDS in protein, using the acceleration parameters found during the search in water. By doing this, the resulting simulation will not have an equal sampling of all of the compounds but only of those with the strongest affinity. An additional production run with the acceleration parameters found during the search in bulk water was performed. The resulting run sampled mostly only 3 of the 11 endstates, *N*-butyl-benzene with 47%, *iso*-butyl-benzene with 27%, and *N*-propyl-benzene with 14% of the total simulation time, while the rest of the compounds had sampling times below 3%. The three most sampled compounds match those that have the lowest Δ*G*_bind_, and the fraction of time that they were sampled also ranks them reasonably based on their experimental free energies (*N*-butyl-benzene −28.03 kJ mol^–1^, *iso*-butyl-benzene −27.24 kJ mol^–1^, *N*-propyl-benzene −27.57 kJ mol^–1^), with the best performing compound appearing in the majority of the simulation, and the next two showing similar sampling times, in agreement with very similar values of Δ*G*_bind_ (less than 0.5 kJ mol^–1^ difference). Finally, the free-energy differences for the top three compounds were computed (*N*-butyl-benzene −30.1 kJ mol^–1^, *iso*-butyl-benzene −27.5 kJ mol^–1^, *N*-propyl-benzene −27.1 kJ mol^–1^), obtaining an RMSE of 1.5 kJ mol^–1^. Note that for this simulation, the system was equilibrated using the benzene-bound structure, which is not one of the best binding compounds. From these results, it seems that AEDS is capable of efficiently screening for the most promising compounds, even if the initial structure is not optimal for these compounds.

## Discussion

In this work, we showed the capabilities of the AEDS technique on a well-studied but challenging system, the L99A mutant of T4 lysozyme.^29−31^ We emphasize the sampling of relevant slow orthogonal degrees of freedom and provide guidelines on how to correctly use this new technique. AEDS showed a significant increase in the sampling of the dihedral transitions of valine 111 in comparison to the simulations in presence of a ligand, a sampling limitation that is known to greatly affect the free energies and that has been extensively studied in the past.^32−37^ All of the previous work in which this valine has been addressed to obtain correct free energies relied on the presence of some kind of bias to sample the appropriate conformations of Val111. For instance, applying an external force to restrain the valine conformation or forcing the transition between its two states.^34^ AEDS did not require a targeted bias and provided free energies at the same level of accuracy as the previous works, with small differences that are inside the expected variance between the force fields used.^50,65^ Furthermore, the overall simulation time required to estimate affinities for all 11 compounds was lower than the simulation times previously used for this system. Including the search runs and the adjustment of the offset, the AEDS simulations were based on 30 ns of simulation time, while other approaches were typically based on simulations from 10 ns to more than 40 ns for every individual pair of compounds. Moreover, the simulation time required for AEDS is even lower if one is only interested in obtaining information on the best binders; for this application, only the 10 ns of production is sufficient. The extra computational cost that AEDS adds to a regular MD simulation is negligible. For the studied system, the AEDS simulations were only about 3% slower than conventional MD simulations, making AEDS an attractive technique to study congeneric series of ligands. Currently, AEDS is only available with the GROMOS engine.^49^ However, an implementation for OpenMM^66^ is planned to be released soon.

Another well-known slow process that is present in this system is the movement of helix F to accommodate bigger ligands.^38−42^ The displacement of this helix is often not addressed in works focused on the free energies, and it seems to have a more modest effect in the final free energies. The average conformation of helix F during the simulation was in between the closed and intermediate states with fluctuations larger than the atom-positional RMSD observed in crystal structures of helix F belonging to the same group.^41^ This suggests that AEDS was able to sample both conformations relevant for the closed and intermediate states. The only helix conformation that was not sampled is the open conformation. This is in agreement with the experimental observation that none of the compounds under study have a preference for this conformation.

AEDS still presents some caveats, especially regarding the learning of appropriate acceleration parameters, which can subsequently lead to insufficient sampling of some of the endstates of interest. The task of obtaining those parameters is simplified thanks to our already published search algorithm.^46^ However, as shown in this work, a further refinement of the obtained offsets can be performed to achieve sufficiently diverse sampling. Another solution is to divide the endstates into subgroups based on their similarity, severely decreasing the complexity of the conformational space that needs to be sampled in a single run, while still being more efficient than any pairwise approach. Furthermore, we showcased another potential use of AEDS for fast virtual screening and hit-to-lead refinement that was not identified before. Using the parameters obtained during the search run in water, one can already determine the top compounds by merely looking at the overall sampling times. For the most promising molecules, reliable free energies are obtained. This fast-screening approach relies only on the (fast converging) search run in water and does not require any further optimization of parameters in the protein.

Despite the mentioned limitations, the analyses showcased in this work can serve to guide the optimization of the acceleration parameters and to assess the reliability of the free-energy values obtained for the different endstates. Furthermore, it demonstrates new possible uses for this technique, including the sampling of orthogonal degrees of freedom and a fast virtual screening approach. Overall, AEDS provides accurate free energies while also enhancing the sampling of relevant events while requiring less total simulation time since multiple endstates can be analyzed from a single simulation. The use of AEDS presents a promising first step to develop a technique capable of studying systems involving slow processes without the need of previous knowledge of the system while also being able to calculate free energies for multiple ligands at the same time.

## Supporting Information Available

Description of applied restraints in the simulation of the convergence of the AEDS parameter search, valine 111 chi1 plots, reweighted atom-positional RMSD values of helix F, Ramachandran plots for every residue of helix F, RMSFs for the backbone atoms of helix F and free-energy values with nonrefined offset parameters (PDF)
Molecular topology files, initial structures, input files, equilibrated coordinate files, and analysis scripts for all relevant simulations (ZIP)

## Notes

All topological information, initial structures, input files, equilibrated coordinate files and analysis scripts are made available in the Supporting Information. All simulations were performed with the GROMOS suite of programs for molecular simulations, which is available free of charge at www.gromos.net.

## References

[ref1] Durrant J. D.; McCammon J. A. Molecular dynamics simulations and drug discovery. BMC Biol. 2011, 9, 71 10.1186/1741-7007-9-71. 22035460 PMC3203851

[ref2] de Vivo M.; Masetti M.; Bottegoni G.; Cavalli A. Role of molecular dynamics and related methods in drug discovery. J. Med. Chem. 2016, 59, 4035–4061. 10.1021/acs.jmedchem.5b01684. 26807648

[ref3] Jorgensen W. L. The many roles of computation in drug discovery. Science 2004, 303, 1813–1818. 10.1126/science.1096361. 15031495

[ref4] King E.; Aitchison E.; Li H.; Luo R. Recent Developments in Free Energy Calculations for Drug Discovery. Front. Mol. Biosci. 2021, 8, 100516 10.3389/fmolb.2021.712085. PMC8387144 34458321

[ref5] Cournia Z.; Allen B.; Sherman W. Relative binding free energy calculations in drug discovery: recent advances and practical considerations. J. Chem. Inf. Model. 2017, 57, 2911–2937. 10.1021/acs.jcim.7b00564. 29243483

[ref6] Meier K.; Bluck J. P.; Christ C. D. Use of Free Energy Methods in the Drug Discovery Industry. In Free Energy Methods in Drug Discovery: Current State and Future Directions, Armacost K. A.; Thompson D. C., Eds.; American Chemical Society, 2021; Vol. 1397, pp 39–66.

[ref7] Zwanzig R. W. High-temperature equation of state by a perturbation method. I. Nonpolar gases. J. Chem. Phys. 1954, 22, 1420–1426. 10.1063/1.1740409.

[ref8] Wang L.; Wu Y.; Deng Y.; Kim B.; Pierce L.; Krilov G.; Lupyan D.; Robinson S.; Dahlgren M. K.; Greenwood J.; Romero D. L.; Masse C.; Knight J. L.; Steinbrecher T.; Beuming T.; Damm W.; Harder E.; Sherman W.; Brewer M.; Wester R.; Murcko M.; Frye L.; Farid R.; Lin T.; Mobley D. L.; Jorgensen W. L.; Berne B. J.; Friesner R. A.; Abel R. Accurate and Reliable Prediction of Relative Ligand Binding Potency in Prospective Drug Discovery by Way of a Modern Free-Energy Calculation Protocol and Force Field. J. Am. Chem. Soc. 2015, 137, 2695–2703. 10.1021/ja512751q. 25625324

[ref9] Fratev F.; Sirimulla S. An Improved Free Energy Perturbation FEP+ Sampling Protocol for Flexible Ligand-Binding Domains. Sci Rep 2019, 9, 16829 10.1038/s41598-019-53133-1. 31728038 PMC6856381

[ref10] Kirkwood J. G. Statistical Mechanics of Fluid Mixtures. J. Chem. Phys. 1935, 3, 300–313. 10.1063/1.1749657.

[ref11] de Ruiter A.; Petrov D.; Oostenbrink C. Optimization of Alchemical Pathways Using Extended Thermodynamic Integration. J. Chem. Theory Comput. 2021, 17, 56–65. 10.1021/acs.jctc.0c01170. 33351609 PMC7872317

[ref12] Cournia Z.; Chipot C.; Roux B.; York D. M.; Sherman W. Free Energy Methods in Drug Discovery-Introduction. In Free Energy Methods in Drug Discovery: Current State and Future Directions, Armacost K. A.; Thompson D. C., Eds.; American Chemical Society, 2021; Vol. 1397, pp 1–38.

[ref13] Song L. F.; Merz K. M. Evolution of alchemical free energy methods in drug discovery. J. Chem. Inf. Model. 2020, 60, 5308–5318. 10.1021/acs.jcim.0c00547. 32818371

[ref14] Boresch S.; Tettinger F.; Leitgeb M.; Karplus M. Absolute binding free energies: a quantitative approach for their calculation. J. Phys. Chem. B 2003, 107, 9535–9551. 10.1021/jp0217839.

[ref15] Shirts M. R.; Pande V. S. Comparison of efficiency and bias of free energies computed by exponential averaging, the Bennett acceptance ratio, and thermodynamic integration. J. Chem. Phys. 2005, 122, 144107 10.1063/1.1873592. 15847516

[ref16] Christ C. D.; Mark A. E.; van Gunsteren W. F. Basic ingredients of free energy calculations: a review. J. Comput. Chem. 2009, 31, 1569–1582. 10.1002/jcc.21450. 20033914

[ref17] Chodera J. D.; Mobley D. L.; Shirts M. R.; Dixon R. W.; Branson K.; Pande V. S. Alchemical free energy methods for drug discovery: progress and challenges. Curr. Opin. Struct. Biol. 2011, 21, 150–160. 10.1016/j.sbi.2011.01.011. 21349700 PMC3085996

[ref18] Ojeda-May P.; Mushtaq A. U.; Rogne P.; Verma A.; Ovchinnikov V.; Grundström C.; Dulko-Smith B.; Sauer U. H.; Wolf-Watz M.; Nam K. Dynamic Connection between Enzymatic Catalysis and Collective Protein Motions. Biochemistry 2021, 60, 2246–2258. 10.1021/acs.biochem.1c00221. 34250801 PMC8297476

[ref19] Bhati A. P.; Wan S.; Wright D. W.; Coveney P. Rapid, accurate, precise, and reliable relative free energy prediction using ensemble based thermodynamic integration. J. Chem. Theory Comput. 2017, 13, 210–222. 10.1021/acs.jctc.6b00979. 27997169

[ref20] Fu H.; Zhang H.; Chen H.; Shao X.; Chipot C.; Cai W. Zooming across the free-energy landscape: shaving barriers, and flooding valleys. J. Phys. Chem. Lett. 2018, 9, 4738–4745. 10.1021/acs.jpclett.8b01994. 30074802

[ref21] Schauperl M.; Czodrowski P.; Fuchs J. E.; Huber R. G.; Waldner B. J.; Podewitz M.; Kramer C.; Liedl K. R. Binding pose flip explained via enthalpic and entropic contributions. J. Chem. Inf. Model. 2017, 57, 345–354. 10.1021/acs.jcim.6b00483. 28079371 PMC5331458

[ref22] Wahl J.; Smieško M. Assessing the predictive power of relative binding free energy calculations for test cases involving displacement of binding site water molecules. J. Chem. Inf. Model. 2019, 59, 754–765. 10.1021/acs.jcim.8b00826. 30640456

[ref23] Sadiq S. K.; Mazzeo M. D.; Zasada S. J.; Manos S.; Stoica I.; Gale C.; Watson S. J.; Kellam P.; Brew S.; Coveney P. Patient-specific simulation as a basis for clinical decision-making. Philos. Trans. R. Soc., A 2008, 366, 3199–3219. 10.1098/rsta.2008.0100. 18573758

[ref24] Wang L.; Friesner R. A.; Berne B. J. Replica exchange with solute scaling: a more efficient version of replica exchange with solute tempering (REST2). J. Phys. Chem. B 2011, 115, 9431–9438. 10.1021/jp204407d. 21714551 PMC3172817

[ref25] Graf M. M. H.; Maurer M.; Oostenbrink C. Free-energy calculations of residue mutations in a tripeptide using various methods to overcome inefficient sampling. J. Comput. Chem. 2016, 37, 2597–2605. 10.1002/jcc.24488. 27634475 PMC5082540

[ref26] Kaus J. W.; McCammon J. A. Enhanced ligand sampling for relative protein–ligand binding free energy calculations. J. Phys. Chem. B 2015, 119, 6190–6197. 10.1021/acs.jpcb.5b02348. 25906170 PMC4442669

[ref27] Brotzakis Z. F.; Limongelli V.; Parrinello M. Accelerating the calculation of protein–ligand binding free energy and residence times using dynamically optimized collective variables. J. Chem. Theory Comput. 2019, 15, 743–750. 10.1021/acs.jctc.8b00934. 30537822

[ref28] Hritz J.; Oostenbrink C. Efficient free energy calculations for compounds with multiple stable conformations separated by high energy barriers. J. Phys. Chem. B 2009, 113, 12711–12720. 10.1021/jp902968m. 19722597

[ref29] Morton A.; Matthews B. W. Specificity of ligand binding in a buried nonpolar cavity of T4 lysozyme: linkage of dynamics and structural plasticity. Biochemistry 1995, 34, 8576–8588. 10.1021/bi00027a007. 7612599

[ref30] Morton A.; Baase W. A.; Matthews B. W. Energetic origins of specificity of ligand binding in an interior nonpolar cavity of T4 lysozyme. Biochemistry 1995, 34, 8564–8575. 10.1021/bi00027a006. 7612598

[ref31] Bouvignies G.; Vallurupalli P.; Hansen D. F.; Correia B. E.; Lange O.; Bah A.; Vernon R. M.; Dahlquist F. W.; Baker D.; Kay L. E. Solution structure of a minor and transiently formed state of a T4 lysozyme mutant. Nature 2011, 477, 111–114. 10.1038/nature10349. 21857680 PMC3706084

[ref32] Deng Y.; Roux B. Calculation of standard binding free energies: Aromatic molecules in the T4 lysozyme L99A mutant. J. Chem. Theory Comput. 2006, 2, 1255–1273. 10.1021/ct060037v. 26626834

[ref33] Mobley D. L.; Graves A. P.; Chodera J. D.; McReynolds A. C.; Shoichet B. K.; Dill K. A. Predicting absolute ligand binding free energies to a simple model site. J. Mol. Biol. 2007, 371, 1118–1134. 10.1016/j.jmb.2007.06.002. 17599350 PMC2104542

[ref34] Mobley D. L.; Chodera J. D.; Dill K. A. Confine-and-release method: obtaining correct binding free energies in the presence of protein conformational change. J. Chem. Theory Comput. 2007, 3, 1231–1235. 10.1021/ct700032n. 18843379 PMC2562444

[ref35] Jiang W.; Roux B. Free energy perturbation Hamiltonian replica-exchange molecular dynamics (FEP/H-REMD) for absolute ligand binding free energy calculations. J. Chem. Theory Comput. 2010, 6, 2559–2565. 10.1021/ct1001768. 21857813 PMC3157700

[ref36] Khavrutskii I. V.; Wallqvist A. Improved binding free energy predictions from single-reference thermodynamic integration augmented with hamiltonian replica exchange. J. Chem. Theory Comput. 2011, 7, 3001–3011. 10.1021/ct2003786. 22046108 PMC3200539

[ref37] Jiang W.; Thirman J.; Jo S.; Roux B. Reduced free energy perturbation/Hamiltonian replica exchange molecular dynamics method with unbiased alchemical thermodynamic axis. J. Phys. Chem. B 2018, 122, 9435–9442. 10.1021/acs.jpcb.8b03277. 30253098 PMC6339808

[ref38] Baase W. A.; Liu L.; Tronrud D. E.; Matthews B. W. Lessons from the lysozyme of phage T4. Protein Sci. 2010, 19, 631–641. 10.1002/pro.344. 20095051 PMC2867005

[ref39] Mobley D. L.; Gilson M. K. Predicting binding free energies: frontiers and benchmarks. Annu. Rev. Biophys. 2017, 46, 531 10.1146/annurev-biophys-070816-033654. 28399632 PMC5544526

[ref40] Boyce S. E.; Mobley D. L.; Rocklin G. J.; Graves A. P.; Dill K. A.; Shoichet B. K. Predicting ligand binding affinity with alchemical free energy methods in a polar model binding site. J. Mol. Biol. 2009, 394, 747–763. 10.1016/j.jmb.2009.09.049. 19782087 PMC2788029

[ref41] Merski M.; Fischer M.; Balius T. E.; Eidam O.; Shoichet B. K. Homologous ligands accommodated by discrete conformations of a buried cavity. Proc. Natl. Acad. Sci. U.S.A. 2015, 112, 5039–5044. 10.1073/pnas.1500806112. 25847998 PMC4413287

[ref42] Lim N. M.; Wang L.; Abel R.; Mobley D. L. Sensitivity in binding free energies due to protein reorganization. J. Chem. Theory Comput. 2016, 12, 4620–4631. 10.1021/acs.jctc.6b00532. 27462935 PMC5021633

[ref43] Oostenbrink C. Free Energy Calculations from One-Step Perturbations. In Computational Drug Discovery and Design; Springer: New York, NY, 2012; pp 487–499. 10.1007/978-1-61779-465-0_28 22183553

[ref44] Christ C. D.; van Gunsteren W. F. Enveloping distribution sampling: A method to calculate free energy differences from a single simulation. J. Chem. Phys. 2007, 126, 184110 10.1063/1.2730508. 17508795

[ref45] Perthold J. W.; Oostenbrink C. Accelerated enveloping distribution sampling: Enabling sampling of multiple end states while preserving local energy minima. J. Phys. Chem. B 2018, 122, 5030–5037. 10.1021/acs.jpcb.8b02725. 29669415

[ref46] Perthold J. W.; Petrov D.; Oostenbrink C. Toward automated free energy calculation with accelerated enveloping distribution sampling (A-EDS). J. Chem. Inf. Model. 2020, 60, 5395–5406. 10.1021/acs.jcim.0c00456. 32492343 PMC7686955

[ref47] Miao Y.; Feher V. A.; McCammon J. A. Gaussian accelerated molecular dynamics: unconstrained enhanced sampling and free energy calculation. J. Chem. Theory Comput. 2015, 11, 3584–3595. 10.1021/acs.jctc.5b00436. 26300708 PMC4535365

[ref48] Anandakrishnan R.; Aguilar B.; Onufriev Av. H++ 3.0: automating p K prediction and the preparation of biomolecular structures for atomistic molecular modeling and simulations. Nucleic Acids Res. 2012, 40, W537–W541. 10.1093/nar/gks375. 22570416 PMC3394296

[ref49] Schmid N.; Christ C. D.; Christen M.; Eichenberger A. P.; van Gunsteren W. F. Architecture, implementation and parallelisation of the GROMOS software for biomolecular simulation. Comput. Phys. Commun. 2012, 183, 890–903. 10.1016/j.cpc.2011.12.014.

[ref50] Reif M. M.; Hünenberger P. H.; Oostenbrink C. New interaction parameters for charged amino acid side chains in the GROMOS force field. J. Chem. Theory Comput. 2012, 8, 3705–3723. 10.1021/ct300156h. 26593015

[ref51] Malde A. K.; Zuo L.; Breeze M.; Stroet M.; Poger D.; Nair P. C.; Oostenbrink C.; Mark A. E. An automated force field topology builder (ATB) and repository: version 1.0. J. Chem. Theory Comput. 2011, 7, 4026–4037. 10.1021/ct200196m. 26598349

[ref52] Petrov D. Perturbation Free-Energy Toolkit: An Automated Alchemical Topology Builder. J. Chem. Inf. Model. 2021, 61, 4382–4390. 10.1021/acs.jcim.1c00428. 34415755 PMC8479811

[ref53] Michel J.; Essex J. W. Prediction of protein–ligand binding affinity by free energy simulations: assumptions, pitfalls and expectations. J. Comput.-Aided Mol. Des. 2010, 24, 639–658. 10.1007/s10822-010-9363-3. 20509041

[ref54] Mey A. S. J. S.; Allen B. K.; Bruce Macdonald H. E.; Chodera J. D.; Hahn D. F.; Kuhn M.; Michel J.; Mobley D. L.; Naden L. N.; Prasad S.; Rizzi A.; Scheen J.; Shirts M. R.; Tresadern G.; Xu H. Best practices for alchemical free energy calculations [article v1. 0]. Living J. Comput. Mol. Sci. 2020, 2, 18378 10.33011/livecoms.2.1.18378. 34458687 PMC8388617

[ref55] Berendsen H. J. C.; Postma J. P. M.; van Gunsteren W. F.; Hermans J. Interaction Models for Water in Relation to Protein Hydration. In Intermolecular Forces, Springer: Dordrecht, 1981; pp 331–342.

[ref56] Eichenberger A. P.; Allison J. R.; Dolenc J.; Geerke D. P.; Horta B. A. C.; Meier K.; Oostenbrink C.; Schmid N.; Steiner D.; Wang D.; van Gunsteren W. F. GROMOS++ software for the analysis of biomolecular simulation trajectories. J. Chem. Theory Comput. 2011, 7, 3379–3390. 10.1021/ct2003622. 26598168

[ref57] Martyna G. J.; Klein M. L.; Tuckerman M. Nosé–Hoover chains: The canonical ensemble via continuous dynamics. J. Chem. Phys. 1992, 97, 2635–2643. 10.1063/1.463940.

[ref58] Berendsen H. J. C.; Postma J. P. M.; van Gunsteren W. F.; DiNola A.; Haak J. R. Molecular dynamics with coupling to an external bath. J. Chem. Phys. 1984, 81, 3684–3690. 10.1063/1.448118.

[ref59] Ryckaert J.-P.; Ciccotti G.; Berendsen H. J. C. Numerical integration of the cartesian equations of motion of a system with constraints: molecular dynamics of n-alkanes. J. Comput. Phys. 1977, 23, 327–341. 10.1016/0021-9991(77)90098-5.

[ref60] Tironi I. G.; Sperb R.; Smith P. E.; van Gunsteren W. F. A generalized reaction field method for molecular dynamics simulations. J. Chem. Phys. 1995, 102, 5451–5459. 10.1063/1.469273.

[ref61] Heinz T. N.; van Gunsteren W. F.; Hünenberger P. H. Comparison of four methods to compute the dielectric permittivity of liquids from molecular dynamics simulations. J. Chem. Phys. 2001, 115, 1125–1136. 10.1063/1.1379764.

[ref62] Chodera J. D.; Swope W. C.; Pitera J. W.; Seok C.; Dill K. A. Use of the weighted histogram analysis method for the analysis of simulated and parallel tempering simulations. J. Chem. Theory Comput. 2007, 3, 26–41. 10.1021/ct0502864. 26627148

[ref63] Oostenbrink C.; van Gunsteren W. F. Free energies of ligand binding for structurally diverse compounds. Proc. Natl. Acad. Sci. U.S.A. 2005, 102, 6750–6754. 10.1073/pnas.0407404102. 15767587 PMC1100734

[ref64] Hintze B. J.; Lewis S. M.; Richardson J. S.; Richardson D. C. Molprobity’s ultimate rotamer-library distributions for model validation. Proteins 2016, 84, 1177–1189. 10.1002/prot.25039. 27018641 PMC4983197

[ref65] Lee J.; Cheng X.; Swails J. M.; Yeom M. S.; Eastman P. K.; Lemkul J. A.; Wei S.; Buckner J.; Jeong J. C.; Qi Y.; Jo S.; Pande V. S.; Case D. A.; Brooks C. L.; MacKerell A. D.; Klauda J. B.; Im W. CHARMM-GUI input generator for NAMD, GROMACS, AMBER, OpenMM, and CHARMM/OpenMM simulations using the CHARMM36 additive force field. J. Chem. Theory Comput. 2016, 12, 405–413. 10.1021/acs.jctc.5b00935. 26631602 PMC4712441

[ref66] Eastman P.; Swails J.; Chodera J. D.; McGibbon R. T.; Zhao Y.; Beauchamp K. A.; Wang L.-P.; Simmonett A. C.; Harrigan M. P.; Stern C. D.; Wiewiora R. P.; Brooks B. R.; Pande V. S. OpenMM 7: Rapid development of high performance algorithms for molecular dynamics. PLoS Comput. Biol. 2017, 13, e1005659 10.1371/journal.pcbi.1005659. 28746339 PMC5549999

