# Supplementary material for: Accelerated Enveloping Distribution Sampling (AEDS) Allows for Efficient Sampling of Orthogonal Degrees of Freedom
Source: J Chem Inf Model. 2022 Dec 13;63(1):197–207. doi: 10.1021/acs.jcim.2c01272 (PMC9832482; doi:10.1021/acs.jcim.2c01272)
Supplement: Supplementary file 1 — ci2c01272_si_001.pdf [file ci2c01272_si_001.pdf]

Supporting information

Accelerated Enveloping Distribution Sampling (AEDS) allows for efficient sampling of orthogonal degrees of freedom

Oriol Gracia Carmona[a] and Chris Oostenbrink[a]*

a. Institute for Molecular Modeling and Simulation, Department of Material Sciences and Process Engineering, University of Natural Resources and Life Sciences, Vienna. Muthgasse 18, 1190 Vienna Austria

S1. Restraints used

To avoid the ligand from diffusing away from the active site during the accelerated enveloping distributions sampling (AEDS) molecular dynamics simulations, weak distance and dihedral restraints were used. The restraints were defined such that they did not involve the parts of the protein motion that is explicitly analyzed in the simulations, to avoid biasing those results. A harmonic dihedral restraint with reference value of 117.4 degree is defined using the Cα and carbonyl C of Ala99 and the carbon atoms 3a and 7a of the ligand, using the indene for numbering. The force constant used was 0.0508 kJ mol^{-1} rad^{-2}.

For the harmonic distance restraint, the center of geometry of the benzene ring of the ligand and the center of geometry of the backbone of Ala99 were used, with a restraining length of 0.507 nm and a force constant of 125 kJ mol^{-1} nm^{-2}. A graphical representation of the atoms involved in the restraining can be found in figure S1.

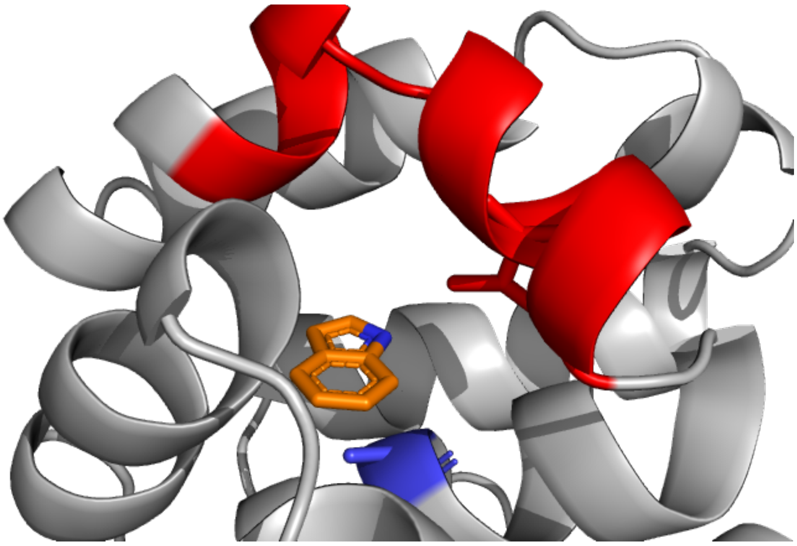

Figure S1: Binding site of the T4L L99A lysozyme with the ligand Indole as reference (pdb 185L) in orange [1]. In red are the protein regions whose motions are relevant for the binding of the ligands (valine 111 and helix F), in blue is the residue alanine 99 whose backbone was used to define the restrains.

S2. AEDS search convergence

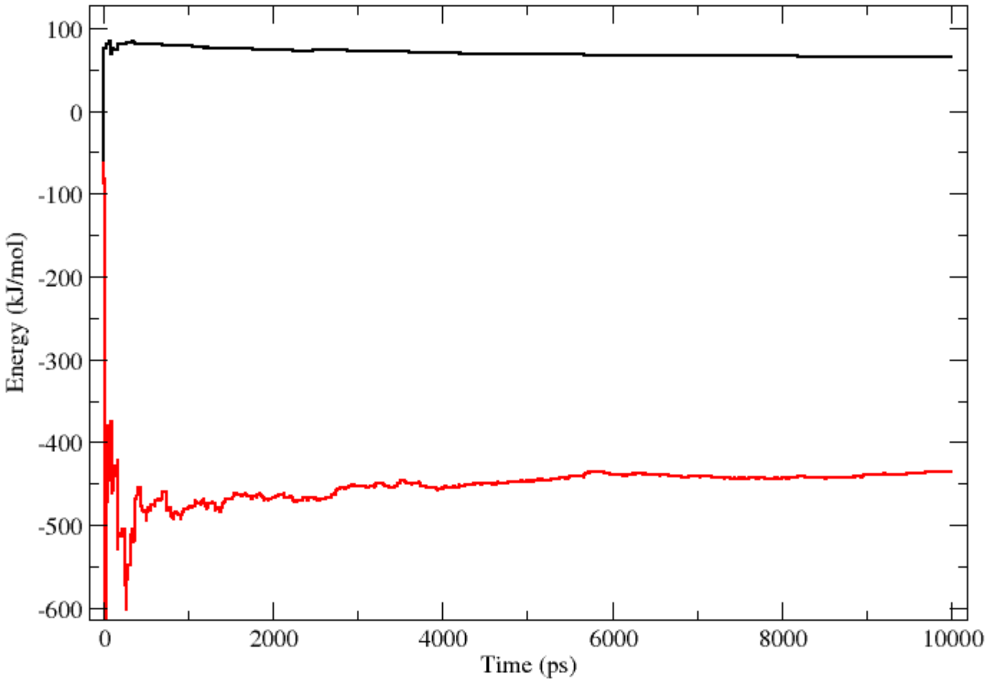

Figure S2: Convergence plots of the E_{max} (black line) and E_{min} (red line) over the 10 ns AEDS search run of the ligands in solution.

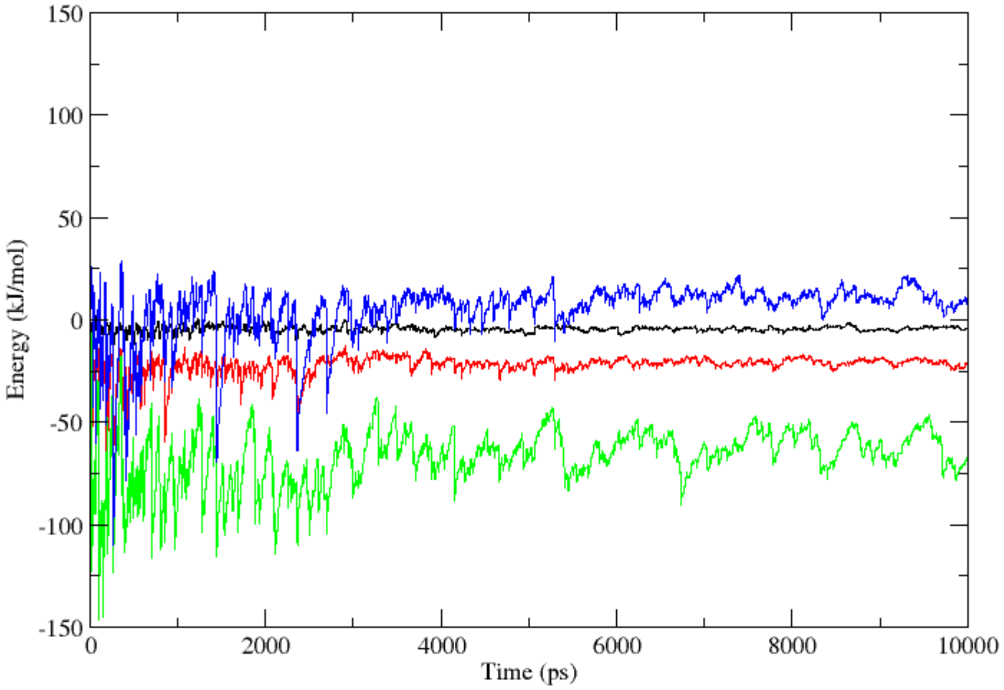

Figure S3: Convergence plots of the offsets of iIndole (black), Benzofurane (red), benzene (green) and ortho-xylene (blue) over the 10 ns AEDS search run of the ligands in solution.

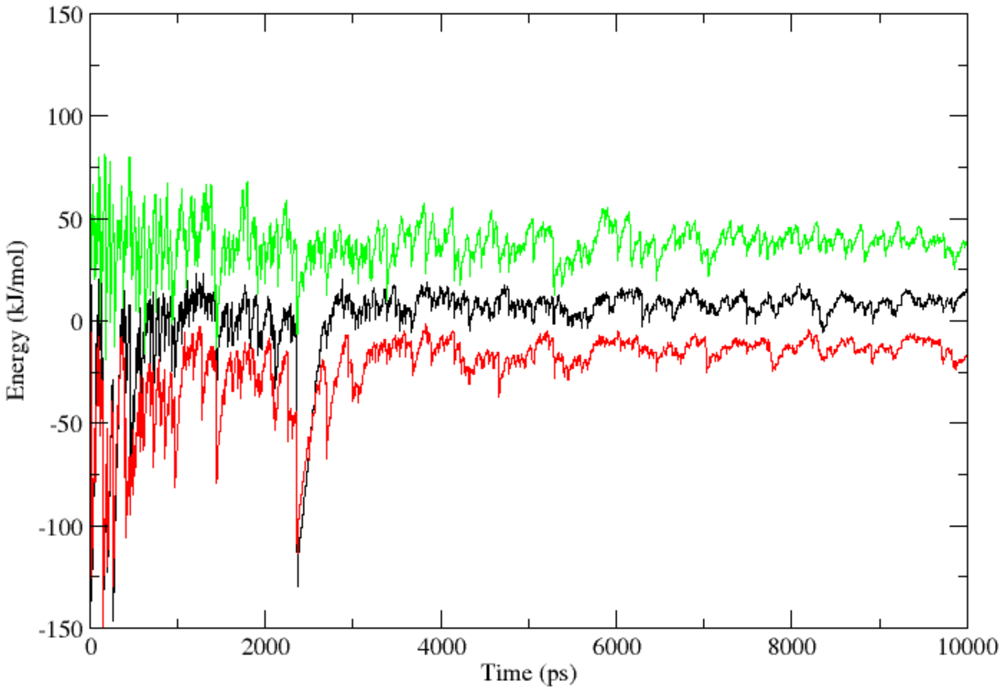

Figure S4: Convergence plots of the offsets of para-xylene (black), toluene (red) and n-propyl-benzene (green) over the 10 ns AEDS search run of the ligands in solution.

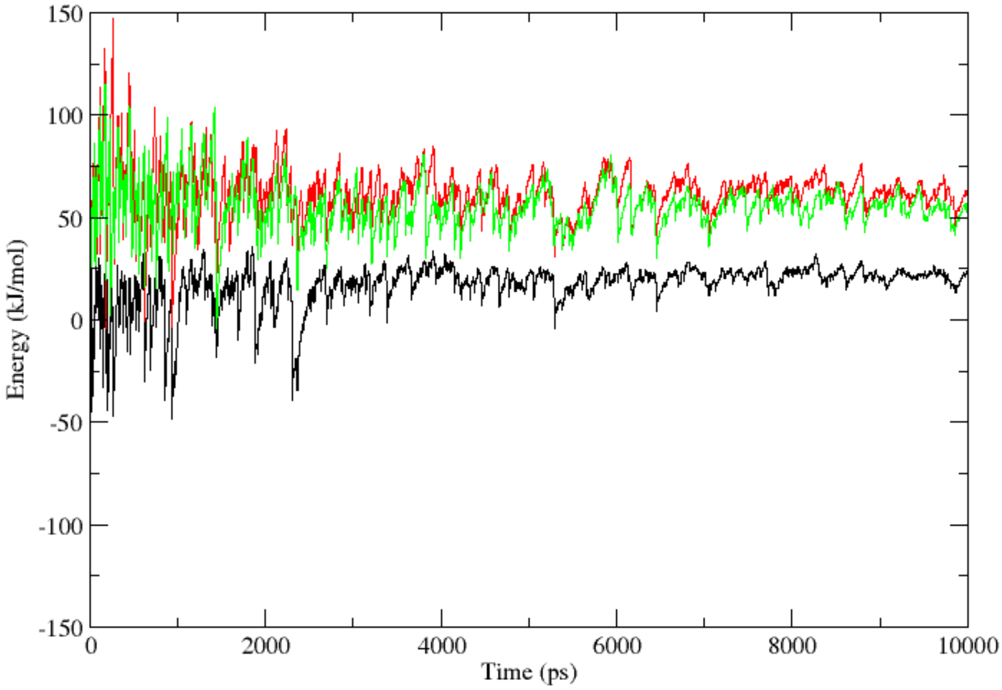

Figure S5: Convergence plots of the offsets of ethylbenzene (black), isobutylbenzene (red) and n-butyl-benzene (green) over the 10 ns AEDS search run of the ligands in solution.

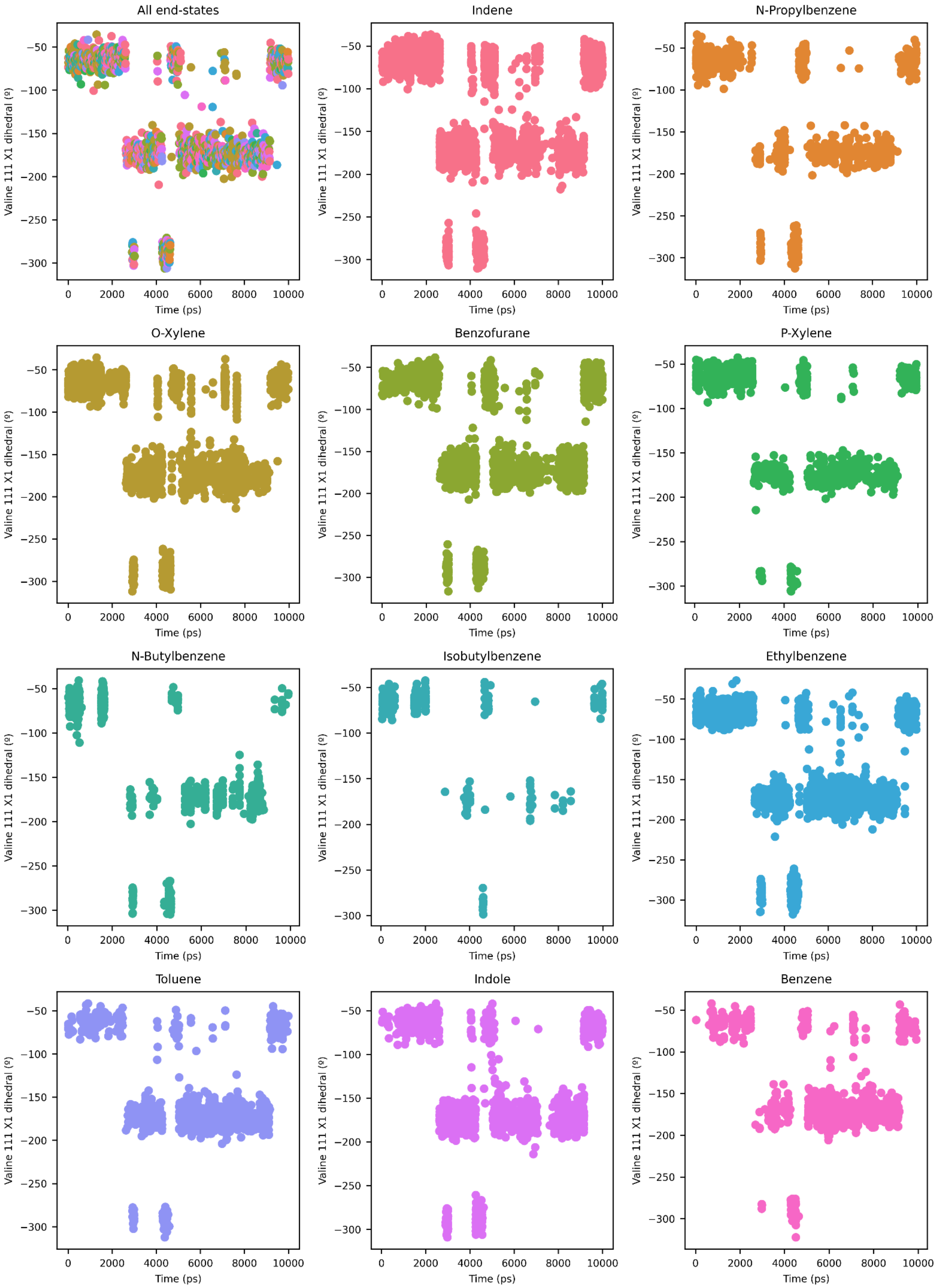

Figure S6: Valine 111 chi1 dihedral plots for all the endstates (first panel) and for each of the endstates by separate.

S3. Reweighted RMSD to closed helix F conformation:

	RMSD closed	RMSD intermediate
Indene	0.076	0.124
Indole	0.070	0.126
Benzofurane	0.080	0.129
Benzene	0.075	0.127
Ortho-xylene	0.071	0.123
Para-xylene	0.058	0.123
Toluene	0.066	0.126
N-propyl-benzene	0.052	0.133
Ethylbenzene	0.064	0.127
Isobutylbenzene	0.051	0.132
N-butyl-Benzene	0.052	0.138

Table S1: Reweighted RMSD profile (in nm) of the difference endstates with respect to the reference helix F for the closed and intermediate conformations [2].

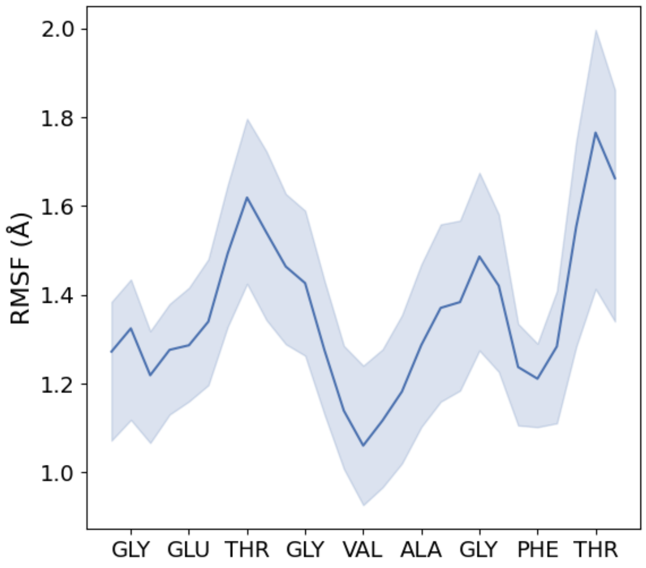

Figure S7. Root mean square fluctuation (RMSF) of the backbone atoms of the helix F during the AEDS simulations. The shadowed area 6represents the 95% confidence interval estimated from 4 independent simulations. All the atoms of the helix backbone fluctuated more than the differences observed within any of the conformations (~1 Å).

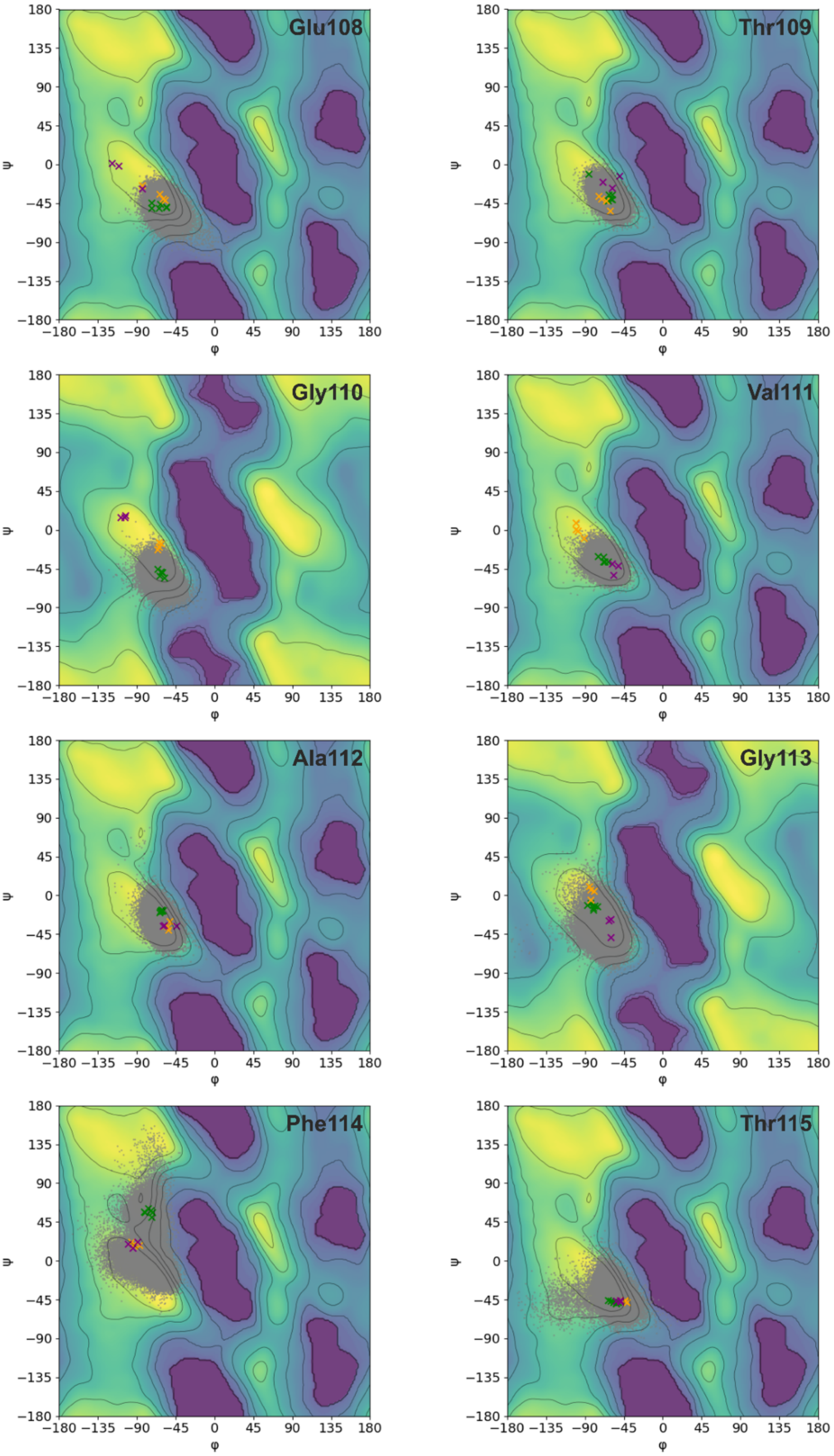

Figure S8. Ramachandran plots for all the residues of the helix F. The green crosses depict the dihedrals for the closed conformation, the orange crosses for the intermediate conformation and the purple crosses for the open conformation, extracted from [2]. The grey dots represent the dihedrals observed during the AEDS simulations. The background of the figures depicts the expected dihedral distribution for the Ramachandran plot of that residue [3], with yellow being the most probable configurations and purple the least probable ones.

S4. Free energies of run with unrefined offsets:

	ΔG_{bind} (kJ/mol)	
	Exp ΔG	AEDS non-optimal offsets
Indene	-22.84 (0.04)	-20.6
Indole	-20.46 (0.25)	-22.1
Benzofurane	-22.84 (0.12)	-16.6
Benzene	-21.71 (0.67)	-19.5
Ortho-xylene	-19.25 (0.25)	-19.5
Para-xylene	-19.54 (0.25)	-23.5
Toluene	-22.97 (0.25)	-22.9
N-propyl-benzene	-27.57 (0.08)	-31.5
Ethylbenzene	-24.10 (0.29)	-21.7
Isobutylbenzene	-27.24 (0.25)	-28.0
N-butyl-Benzene	-28.03 (0.08)	-30.7
RMSE	-	3.1
Slope	-	1.17
R^2	-	0.59

Table S2: Free energy differences in kJ/mol estimated from the AEDs run with non-optimal offsets, with their standard error in brackets, their respective root mean square error (RMSE), slope of the linear regression and correlation coefficient (R2). Experimental values are extracted from [5,6]. The AEDS free energies are calculated as ΔΔG from the reference state and then shifted to the experimental values, the lost degree of freedom is accounted for when calculating the RMSE.

References

1 Morton, A.; Matthews, B. W. Specificity of ligand binding in a buried nonpolar cavity of T4 lysozyme: linkage of dynamics and structural plasticity. *Biochemistry* **1995**, *34*, 8576-8588.

2 Merski, M.; Fischer, M.; Balius, T. E.; Eidam, O.; Shoichet, B. K. Homologous ligands accommodated by discrete conformations of a buried cavity. *Proc. Natl. Acad. Sci. U.S.A.* **2015**, *112,* 5039-5044.

3 Hintze, B. J.; Lewis, S. M.; Richardson, J. S.; Richardson, D. C. Molprobity's ultimate rotamer-library distributions for model validation. *Proteins* **2016**, *84*, 1177–1189.

4 Morton, A., Baase, W. A., & Matthews, B. W. Energetic origins of specificity of ligand binding in an interior nonpolar cavity of T4 lysozyme. *Biochemistry* **1995**, *34*, 8564-8575.

5 Mobley, D. L.; Graves, A. P.; Chodera, J. D.; McReynolds, A. C.; Shoichet, B. K.; Dill, K. A. Predicting absolute ligand binding free energies to a simple model site. *J. Mol. Biol.* **2007**, *371*, 1118-1134.
